# 1362. Pediatric long COVID subphenotypes: an EHR-based study from the RECOVER program

**DOI:** 10.1093/ofid/ofad500.1199

**Published:** 2023-11-27

**Authors:** Vitaly Lorman, Xing Song, Suchitra Rao, Andrea J Allen, Levon Utidjian, L Charles Bailey

**Affiliations:** Children’s Hospital of Philadelphia, Philadelphia, Pennsylvania; University of Missouri, Columbia, Missouri; University of Colorado School of Medicine, Aurora, Colorado; Children's Hospital of Philadelphia, Philadelphia, Pennsylvania; Children's Hospital of Philadelphia, Philadelphia, Pennsylvania; University of Pennsylvania, Philadelphia, Pennsylvania

## Abstract

**Background:**

Post-acute sequelae of COVID-19 (PASC, or long COVID) has been associated with a wide variety of symptoms, conditions, and body systems; presentation in children has been particularly heterogeneous and it is important that studies of PASC distinguish among clinical presentations, or subphenotypes.

**Methods:**

Due to the lack of a consensus formal definition of PASC, we identified a cohort of patients in the PEDSnet clinical research network EHR data with probable PASC by selecting SARS-CoV-2 positive patients who had diagnoses of PASC or PASC-associated conditions during the 28 to 179 day post-acute period following infection. To identify subphenotypes, we used clinical concept embedding methods summarized in Figure 1. To evaluate differentiation of clusters, we computed proportions of diagnoses in each of 22 groups of PASC-associated features and summarized clusters by demographics and health system utilization over the PASC trajectory. Finally, to evaluate the reproducibility of the subphenotypes, we applied the same process to a cohort of pediatric patients from 24 non-PEDSnet sites in the RECOVER network.

Concept embedding and subphenotype model flowchart

**Figure:**
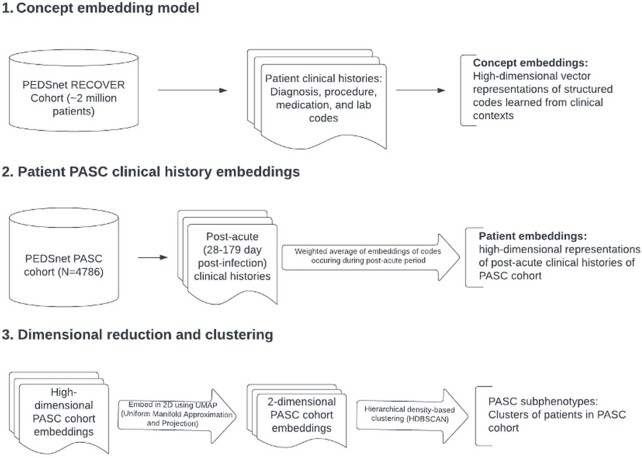

Steps 1 and 2 are modeled on the Phe2Vec automated phenotyping algorithm.

**Results:**

Our approach applied to the PEDSnet cohort of 4786 patients with likely PASC produced 6 clusters of patients, each representing a distinct subphenotype of PASC; prevalence of PASC-associated diagnoses across clusters is summarized in Figure 2. Clusters A, B, and D had clear leading diagnoses, while clusters C and E were were less focused but still maintained a clear theme. Additionally, a sixth cluster (F) was identified as a multisystem subphenotype. Utilization trajectories in each cluster are summarized in Figure 3. The same methods applied to a pediatric cohort of 7370 patients from non-PEDSnet RECOVER sites produced similar respiratory, musculoskeletal, gastroinstestinal, and multisystem clusters. However, the neurologic and cardiopulmonary clusters were merged into one in the non-PEDSnet cohort.

Summary of subphenotypes by prevalence of PASC-associated features

**Figure:**
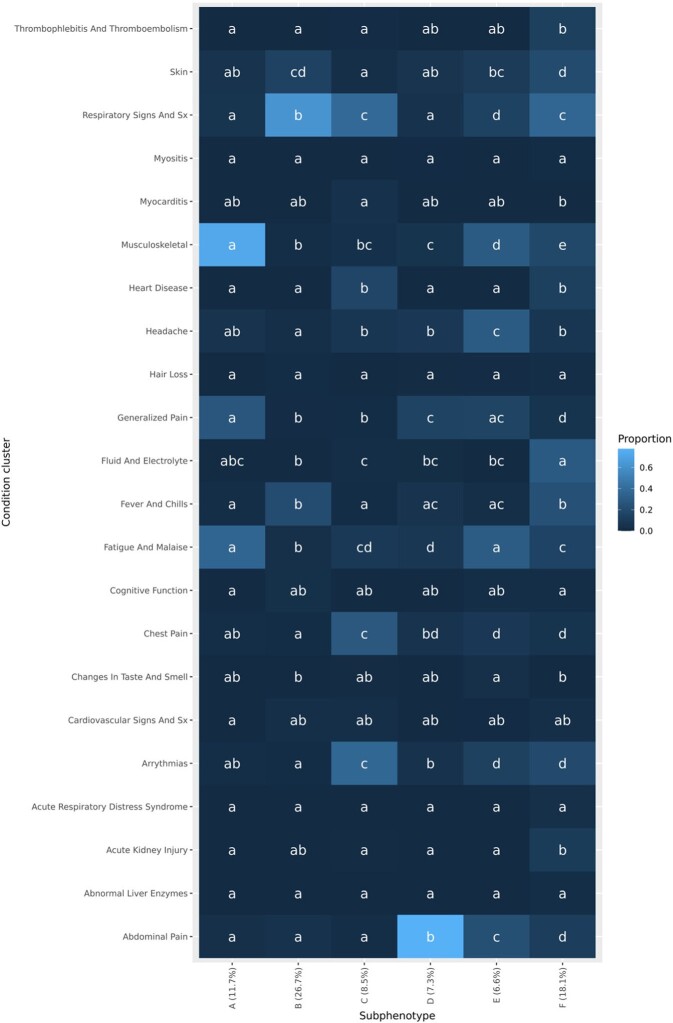

To evaluate differentiation of clusters, we computed proportions of diagnoses in each of 22 groups of PASC-associated features and used Bonferroni-adjusted pairwise chi-squared testing for each combination of PASC-associated feature and pair of clusters, presented in a compact letter display format. For a given PASC-associated feature, two subphenotypes share the same letter when proportions of patients with that feature did not significantly differ between the two subphenotypes.

Health system utilization trajectories by subphenotype

**Figure:**
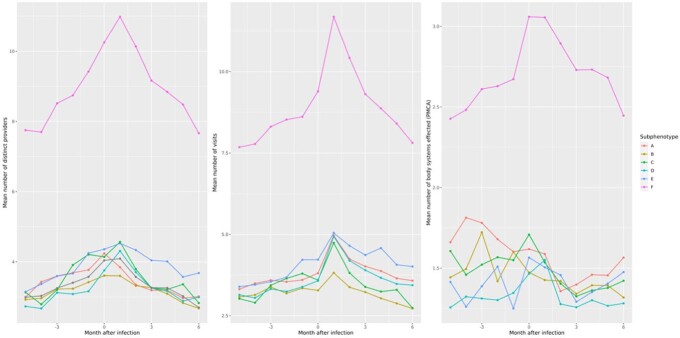

**Conclusion:**

Our study is the first to apply subphenotyping methods to pediatric PASC in EHR data, evaluating a wide range of features across a large, multisite cohort of affected children. The subphenotypes are well-differentiated, clinically plausible, and consistent with early reporting in case studies.

**Disclosures:**

**Suchitra Rao, MBBS, MSCS**, Sequiris: Advisor/Consultant

